# Endodermal Sinus Tumor Presented With Ascites: A Case Report

**DOI:** 10.4021/gr406w

**Published:** 2012-05-20

**Authors:** Ahmet Cumhur Dulger, Huseyin Begenik, Ramazan Esen, Mete Rafet

**Affiliations:** a Yuzuncu Yil University, Medical faculty, Gastroenterology, Van, Turkey; b Yuzuncu Yil University, Medical faculty, Internal Medicine, Van, Turkey

**Keywords:** Endodermal sinus tumor, Low gradient ascites

## Abstract

We report a case of primary endodermal sinus tumor of the omentum which may be the fourth reported case in the English literature. A 19-year-old boy presented with ascites. Analysis of ascites revealed high levels of AFP and CA 125. Laparoscopic biopsy showed endodermal sinus tumour. He was treated with four courses of the BEP regimen (bleomycin, etoposide, cisplatin). The patient was died 2 months after the first appearance of the ascites. Endodermal sinus tumor (EST) is a rare neoplasm which usually arises in the testis or ovary. But extragonadal EST especially located in the abdomen is very rare condition. Clinicians should remain vigilant particularly, when there is a low gradient ascites and are high levels of tumor markers in ascites in young patients.

## Introduction

Endodermal sinus tumor (Yolk sac tumor) is a rare neoplasm characterized by high malignancy given its premature metastasis that is frequent in adolescence. Endodermal sinus tumor (EST) usually arises in a gonad; an extragonadal location is distinctly unusual, and, when it occurs, location in the abdomen is very rare [1]. In this report, we describe a rare case with endodermal sinus tumor and related ascites.

## Case Report

A 19-year-old boy was referred to our hospital because of abdominal dullness and night sweats of 15 days duration. His medical history was normal. Physical examination on admission identified grade III (tense) ascites. In ascites; alpha-fetoprotein (AFP) and CA 125 were determined: AFP was 1200 IU/mL (upper reference value, < 5.5 IU/mL), CA 125 was 500 u/mL (upper reference value, < 16 U/mL) whereas other tumor markers were within the normal range. The serum-to-ascites albumin gradient (SAAG) was calculated as 0.6 g/dL and ascidic adenozine deaminase level was reported as 40 U/L (upper reference value, < 16 U/L).

**Figure 1:**
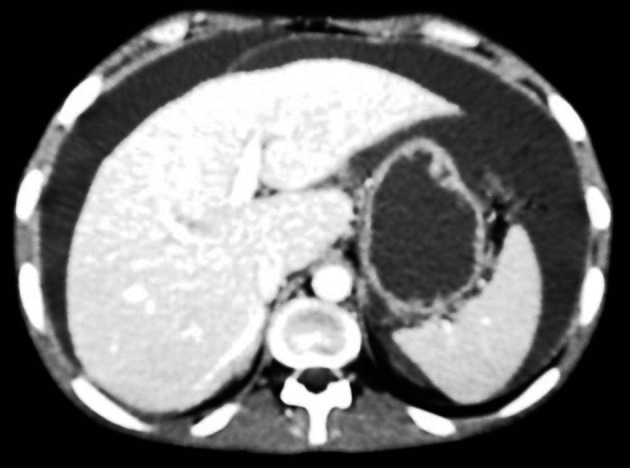
Ascites apperance on CT of the abdomen.

**Figure 2:**
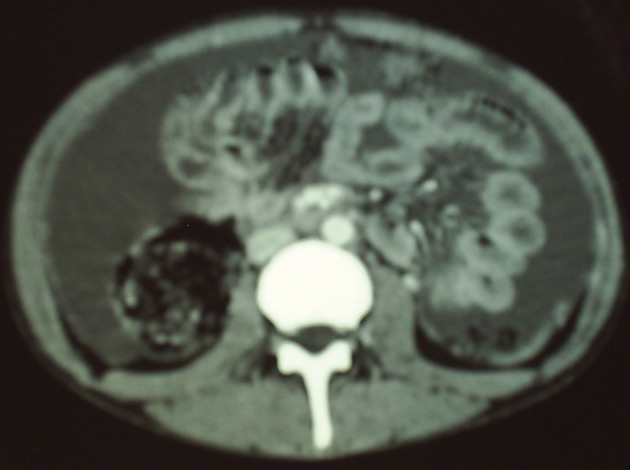
“Omental cake” apperance on abdominal CT.

**Figure 3:**
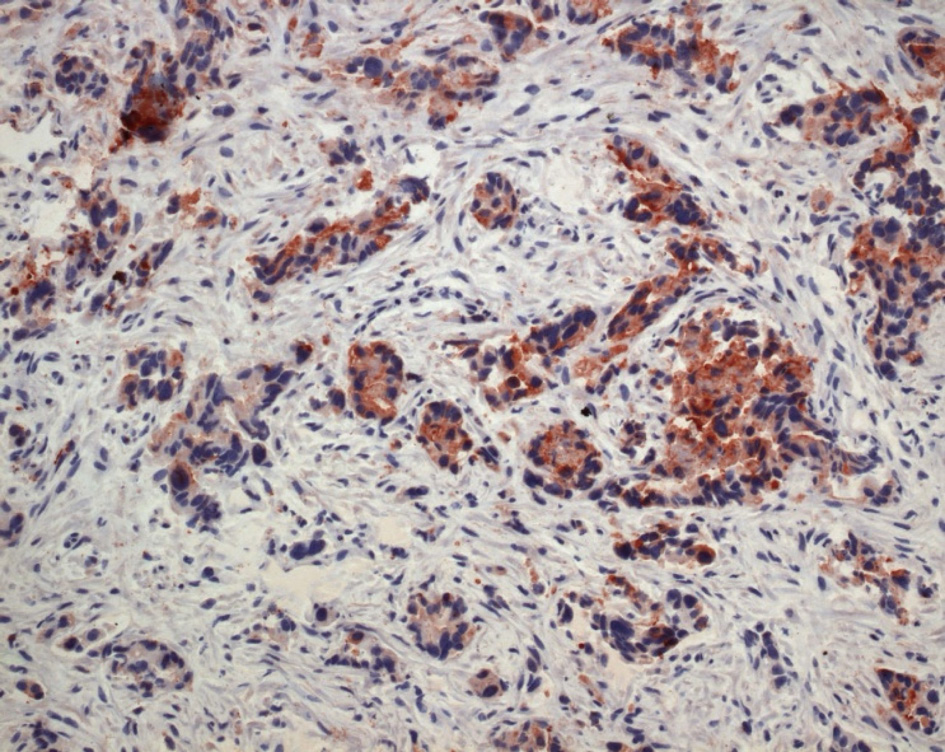
Tumour cells show staining for AFP ( Immunohistochemically x 400 ).

**Figure 4:**
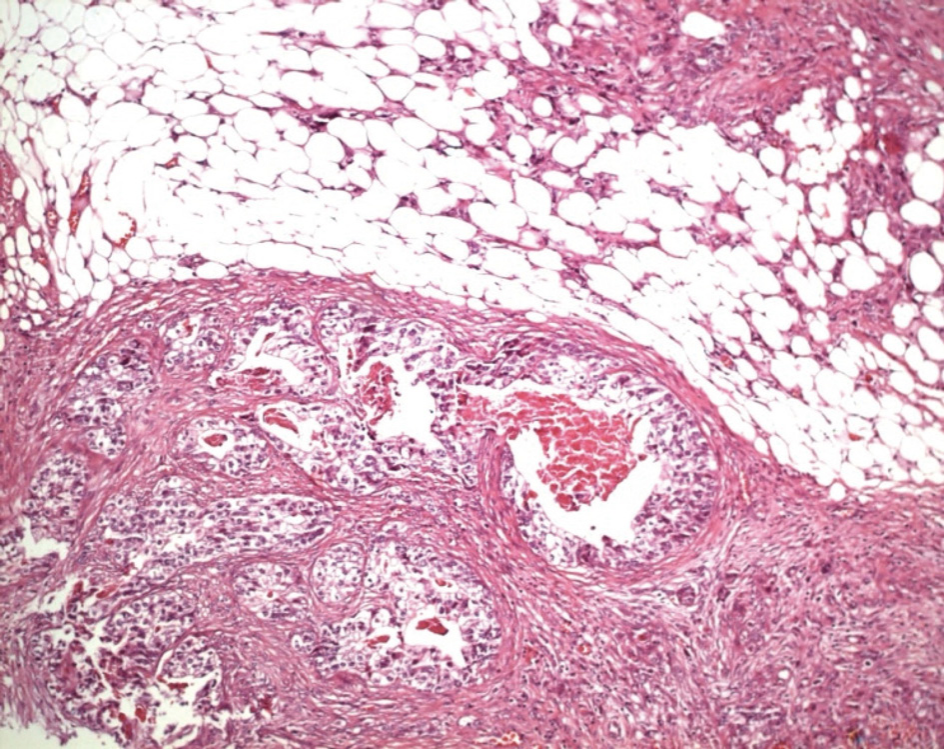
Yolk sac tumour, intestinal type: Numerous dilated angular glands lined by an eosinophilic columnar epithelium were seen (H.E. x 100).

Ultrasonography and CT scan of the abdomen revealed large ascites and omental cake (Fig.1, 2). Additional studies (chest radiograph, colonoscopy and upper gastrointstinal tract endoscopy) were negative for additional masses. There was no evidence of an extraabdominal primary source. A left sided minimal epididymo-orchitis was detected on urinary ultrasonography. The patient underwent laparotomy, which revealed massive peritoneal carcinomatosis and a large amount of ascites. Pathological examination of the peritoneal specimen confirmed the presence of EST and stained for alpha fetoprotein and cytokeratin (Fig. 3, 4).

Post-laparotomy, the patient received combination chemotherapy consisting of cisplatin, etoposide and bleomycin every 3 weeks for a total of 4 cycles. At the end of the treatment his clinical status worsened and the patient refused to take any further treatment including radiotherapy and the patient decided to go to home, 15 days later, he reportedly died due to cardiac arrest.

## Discussion

Testicular neoplasms comprise the most common solid malignancies affecting males between the ages of 15 and 35, although they represent only about 1 percent of all solid tumors in men [2]. The two main categories of testicular tumors are germ cell tumors (GCTs), which account for 95 percent of cases, and sex cord-stromal tumors. The terms "yolk sac tumor" (YST) and "endodermal sinus tumor" are synonymous. Pure YST is the most common malignant testicular GCT in children [3], although teratomas are actually more prevalent in this age group [4]. However, it is rarely seen in the adult. On the other hand, a component of YST is common (about 40 percent) in mixed GCTs arising in adults [5].

Approximately 95 percent of malignant tumors arising in the testis are germ-cell tumors, a term that indicates their origin in primordial germ cells. Germ-cell tumors also occasionally arise in extragonadal primary sites, and their management follows that of testicular germ-cell tumors.

More than 90 percent of patients with newly diagnosed germ-cell tumors are cured, and delay in diagnosis correlates with a higher stage at presentation for treatment [6, 7].

The chief entity in the microscopic differential diagnosis is embryonal carcinoma, with which YST often merges. Cytologically, YST tends to have less nuclear atypia, and sometimes the architectural features are distinctive. Immunohistochemical (IHC) staining for AFP can be helpful, and is generally positive, at least focally, in YST. In contrast, AFP is positive in only a few scattered cells, at most, in embryonal carcinoma. Occasionally, difficulty may be experienced in differentiating YST from seminoma or teratoma. In these cases, IHC staining for cytokeratin and AFP can be useful [8]. The biopsies obtained from laparotomy were displayed typical features of yolk sac tumor (YST) in the presented case.

Testicular cancers usually form a discrete, firm, nontender mass within the testis; however, the entire testis may be enlarged. On physical examination; the left testis and left epididymis were normal and ultrasound examination showed minimal epididymo-orchitis that was not considered as a testiculer cancer.

The serum-to-ascites albumin gradient (SAAG) accurately identifies the presence of portal hypertension and is more useful than the protein-based exudate/transudate concept. The SAAG is easily calculated by subtracting the ascitic fluid albumin value from the serum albumin value, which is obtained on the same day.

The presence of a gradient 1.1 g/dL (11 g/L) indicates that the patient has portal hypertension with 97 percent accuracy. A gradient < 1.1 g/dL (< 11 g/L) indicates that the patient does not have portal hypertension [9]. In this patient, SAAG was 0.6 g/dL. Thus, we ruled out all portal hypertension-related conditions. Malignant ascites is characterised by positive cytology of malignant cells. Compared to ascites caused by cirrhosis, more white blood cells and a higher lactate dehydrogenase level are usually present [10]. Ascites examination of the patient under discussion, revealed markedly raised AFP, CA 125 and LDH levels. It is also necessary in such patients that other conditions known to cause elevated ascitic tumor markers, including tuberculosis, which is endemic in the eastern part of Turkey. So, tuberculosis was ruled out based on near-normal ascidic Adenozine Deaminase level and histopathologic examination of obtained tissues by laparotomy.

Alpha-fetoprotein (AFP) is the major protein of fetal serum but falls to an undetectable level after birth. The primary malignancies associated with AFP elevations are hepatocellular carcinoma and nonseminomatous germ cell tumors. Other gastrointestinal cancers occasionally cause elevations of AFP, but rarely to greater than 1,000 ng/mL [11]. In men with NSGCTs, AFP is produced by yolk sac (endodermal sinus) tumors and, less often, embryonal carcinomas. In addition, up to 80 percent of mediastinal NSGCTs are associated with elevated serum AFP, regardless of histologic subtype [12]. As with beta-hCG, the percentage of tumors with increased serum AFP increases with advancing clinical stage, from 10 to 20 percent of stage I tumors, to 40 to 60 percent of disseminated NSGCTs [13]. Estimation of serum AFP is useful for a diagnosis, monitoring the effectiveness of therapy and detection of recurrence prior to clinical manifestation [14]. On the basis of the locality of the tumor and the levels of tumor markers, the tumor had an advanced stage and had a bad prognosis in our case.

Four cycles of BEP remains the standard of care for the treatment of men who have intermediate- or poor-risk advanced GCTs. Using this approach five-year survival rates for intermediate- and poor-risk patients are 79 and 48 percent, respectively [15].

Blood and ascites were drawn for assessment of tumor markers but his tumor markers remained high after four cycles of chemotherapy.

This case demonstrates that primary YSTs may occur in the omentum and the periton, and they are associated with poor prognosis as in our case. In conclusion, YSTs should be kept in mind in cases with low gradient ascites. Therefore, elevation of tumor markers in ascites should be carefully investigated when evaluating a patient presented with low-gradient ascites.
